# A Cross-Regional Analysis of the COVID-19 Spread during the 2020 Italian Vacation Period: Results from Three Computational Models Are Compared

**DOI:** 10.3390/s20247319

**Published:** 2020-12-19

**Authors:** Luca Casini, Marco Roccetti

**Affiliations:** Department of Computer Science and Engineering, University of Bologna, 40127 Bologna, Italy; luca.casini7@unibo.it

**Keywords:** COVID-19, predictions, computational models, cognitive models, new infections, tourism, Italy

## Abstract

On 21 February 2020, a violent COVID-19 outbreak, which was initially concentrated in Lombardy before infecting some surrounding regions exploded in Italy. Shortly after, on 9 March, the Italian Government imposed severe restrictions on its citizens, including a ban on traveling to other parts of the country. No travel, no virus spread. Many regions, such as those in southern Italy, were spared. Then, in June 2020, under pressure for the economy to reopen, many lockdown measures were relaxed, including the ban on interregional travel. As a result, the virus traveled for hundreds of kilometers, from north to south, with the effect that areas without infections, receiving visitors from infected areas, became infected. This resulted in a sharp increase in the number of infected people; i.e., the daily count of new positive cases, when comparing measurements from the beginning of July to those from at the middle of September, rose significantly in almost all the Italian regions. Upon confirmation of the effect of Italian domestic tourism on the virus spread, three computational models of increasing complexity (linear, negative binomial regression, and cognitive) have been compared in this study, with the aim of identifying the one that better correlates the relationship between Italian tourist flows during the summer of 2020 and the resurgence of COVID-19 cases across the country. Results show that the cognitive model has more potential than the others, yet has relevant limitations. The models should be considered as a relevant starting point for the study of this phenomenon, even if there is still room to further develop them up to a point where they become able to capture all the various and complex spread patterns of this disease.

## 1. Introduction

The SARS-CoV-2 virus that emerged in Wuhan, China, at the end of 2019, causing the current global pandemic, has spread globally extremely quickly. This is unquestionably due to its high infectiveness, but it would not have reached the planetary scale if not for the interconnectedness of the world we live in. As Zhang et al. describe in their paper, the centrality of Wuhan and its multiple transport communication hubs (roadways, railways, airports, and boats) played an important role in the spread of the virus in China in the early days of the infection, showing that those regions that required the least travel time from that city were hit earlier and more fiercely [1].

From there the virus was carried by planes, and by the beginning of March 2020 it reached other Asian countries, and then Europe, Australia, and the Americas. At that point, it was clear that we were heading towards a pandemic, and most countries in the world put in place multiple restrictions to avoid the further spread of the virus, including banning travel (international and, in some countries, even domestic) and closing public places, schools, and offices, as well as introducing strict personal hygiene rules to reduce the possibility of virus transmission.

Put succinctly, these measures were fortunately quite effective in flattening the curve, up to the point that many countries lowered their guard by the end of spring 2020, thinking that the worst was behind them, and ended up risking a second wave in the autumn.

As Tomas Pueyo discusses in his latest article in the New York Times [2], the decision to forbid travel, both international and domestic, was one of the key ingredients in slowing down the contagion and making it manageable. He goes on to analyze, in his article, how the *fence*, that is, the set of containment measures, including travel restrictions and strict monitoring of incoming tourists, had been progressively dismantled with the arrival of summer, making the sacrifices made during the previous months almost useless, as it often took just a few careless travelers to ignite a contagion in a region that was previously unscathed.

Those considerations describe quite well what happened in Italy during the period from February to August 2020. Essentially, after the outbreak of COVID-19 that started at the end of February in Lombardy and then violently hit all of northern Italy, the Italian government imposed a nationwide lockdown (on 9 March) in order to flatten the curve and avoid further spreading of the virus [3]. This measure proved to be effective with the passage of time, and thus the lockdown was gradually lifted at the beginning of May 2020 as the number of new daily active cases steadily declined; all citizens were then allowed to go outside, even when not strictly necessary, and people could gather, for example, in bars and restaurants while still maintaining social distancing. Nonetheless, it was not possible to move freely between regions until 3 June 2020 [4], when the total number of new infected nationwide was averaging between 200 and 300. The count of the new daily infections remained stable until the last week of August 2020, when there was a noticeable uptick that brought the number of new daily cases, on a national level, to over 1200, as described by stored data [4].

This begs the question: What could be the most likely cause of this increase? The answer follows naturally. Considering that the virus is known to take 10–14 days to show its symptoms, this puts the start of this inflation in the first half of August, which is the preferred time of the year for vacation trips in Italy, thus leading to a hypothesis, which is at the center of a national debate, that there was a significant relationship between Italian domestic tourism and the resurgence of the virus during the summer of 2020 [5]. What should also be considered is the fact that, this summer, the most typical Italian tourist destinations were visited mostly by Italian tourists, with little or no contribution by international tourism because many countries had set limits on travel, as well as implementing flying restrictions [6].

This idea of a relationship between Italian domestic tourism and the resurgence of the virus is also reinforced by a careful observation of the number of new daily infections in certain Italian regions that are typical holiday destinations; they were almost virus-free at the beginning of the summer and, in the span of a few week, cases escalated from almost 0 to hundreds, daily. A peculiar example of this phenomenon is the insular region of Sardinia, where the virus had almost disappeared from the island and then suddenly came back in August 2020, surely carried by the tourists that arrived for their summer holidays [7].

After this introduction, which was needed to frame the scenario as a whole, we can now explain the intent behind this study: to investigate the influence of Italian domestic tourism on the resurgence of COVID-19, using the tourist flows between Italian regions as a predictor for the number of new infections in the destination areas.

We began our investigation by looking for confirmation of the most relevant facts that are at the heart of the aforementioned hypothesis. This was done by scrutinizing the daily number of new infection cases during the entire summer period for all of the 20 Italian regions. We utilized a common change point detection method to find the most likely moment of change in the time series of the new daily infections [8]. Results show that the hypothesis of a relevant change in August 2020 had a solid statistical confirmation, which is valid for all the Italian regions included in our study, and it is centered in the last days of August 2020.

This method has also indicated to us that a special focus had to be put on the cumulative number of daily infections each region suffered during the period that started in the middle of August and ended in the middle of September 2020.

What we anticipated can be considered as the first relevant result of our study. We then incorporated a mathematical regression method, which comes as the natural extension of the aforementioned result, into the study: estimating, on a monthly basis, the total amount of new infections using the number of inbound and outbound tourists per region. We did this by taking into careful consideration the peculiarity of the two-week lag before an infected person shows some symptoms of the virus along with a variety of other additional factors that could help explain the dynamics of the pandemic, beside tourism (e.g., regional population density, public sanitary expenditure per region).

Finding a mathematical model able to solve this problem with a good accuracy involves acquiring the capability to predict, in advance, how the spread of COVID-19 progresses with tourists moving across the world. Instead, so far, our models have been able to confirm the hypothesis of a relationship only after that phenomenon had occurred.

Hence, to solve this regression problem, we experimented with three different models, our goal being that of identifying the one that could better represent the relationship between Italian domestic tourism during the summer of 2020 and the resurgence of new COVID-19 cases.

All our approaches were developed with the final intent of comparing the number of people that actually got the infection during the observed period (middle of August to the middle of September, 2020) and the number of infections estimated by the model, for each region. The more accurate the predictions, the more valid the model.

What has to be mentioned is also the fact that, while the number of new infections per Italian region is precisely known for the period of our investigation, the distribution of the 2020 Italian tourist flow is still currently unknown. Given the unavailability of these data, we replaced them in our models with the distribution of the Italian flow of tourists for the same observed period from 2019, as recorded by the Italian Institute of Statistics (ISTAT) [9].

Out of our three different models, we started toying with a naïve one, based on a simple formula that considers, per each region, the number of tourists coming from other regions and the infection rate of those regions. Assuming a constant infection rate for each incoming flow of tourists, the formula counted how many new cases could be attributed to that flow for each given destination area. Finally, all the contributions were totaled to count how many infections had been brought to that region by Italian domestic tourism in that period.

Needless to say, the results we got with this method were a very simplistic estimate of how many of the new infection cases could be attributed to tourism over the considered timespan, per each Italian region.

We then proceeded to a more sophisticated model. Essentially, we fitted a generalized linear model, where the (number of) infections were assumed to be distributed on the basis of a negative binomial distribution and were predicted utilizing a traditional maximum likelihood estimation method [10]. Beside the accuracy of the predictions, the work we have done with this kind of regression analysis is that of providing further evidence in favor of Italian domestic tourism being a factor that determined the resurgence of the virus in many Italian regions during the 2020 summer period.

Finally, dissatisfied with the infection prediction accuracy yielded with the first two models, we applied an artificial neural network to the available set of data to see if introducing a method that can consider non-linear relationships among the features would result in better predictions.

Numerical results have shown that this cognitive model has more predictive potential than the others, yet it has relevant limitations. Nonetheless, even considering the relatively low prediction accuracy of those two models, this is not enough to definitively disqualify them. Instead, they should be considered as a relevant starting point for the study of this phenomenon, even if there is still room to further develop them up to a point where they become able to capture all the various and complex spread patterns of this disease.

Beside the present study, travel, and its role in the spread of COVID-19, is being researched intensively, from various angles. Some works, including ours, focus on the dynamics of people moving from one place to another, while other researchers have focused their efforts on studying the way the virus can be transmitted in certain environments pertaining to travel, such as trains and planes [11,12]. For example, Krisztin et al. used econometric models to study how cross-country air travel played an important role in the early spread of the virus between European countries [13]. However, we shall not delve any deeper into this subject as this would take us too far afield into the background behind our approach.

Rather, it is more interesting to note that our work focuses on Italian data and, at the moment of writing, there seems to be no published work that focuses exclusively on this country, investigating the influence of tourism on the spread of the virus. Indeed, there have been a number of relevant publications that address a similar topic but only in the context of international travel or domestic travel in other nations than Italy.

In this respect, relevant is the work done by Dasgupta and Wheeler, who created a model that tries to estimate how the number of infections evolved as a function of people’s interaction and the COVID-19 infection rates, registered in different geographical areas [14]. This kind of model heavily depends on the geographical distance between areas and, as such, it draws upon a well-known migration modeling technique termed gravity modeling, that is, essentially, a type of regression used to model international migration flows; where, in this specific case, the probability of bringing the infection to a given area has been subjected to an exponential decay depending, in turn, on the relative distance as a reminiscence of the physical gravitational concept of mass.

Additionally, the already mentioned work by Zhang et al. used a kind of gravity model based on the number of infections, which performed a regression analysis of various means of transport in order to investigate how relevant their contribution to the spread of the virus was [1]. But this kind of papers will not be analyzed here because, as already stated, the study of the contribution to the spread of the virus brought by different modes of transport is out of the scope of our investigations.

Instead, in the same vein as [13], Farzanegan et al. studied the role played by tourism in the spread of the virus between countries across the world by using regression analysis to verify the relevance of tourist flows on the total number of infected people in conjunction with other factors, such as, for example, population density and aging, healthcare expenditures, and others [15]. Similarly, Falk and Hangsten have studied how alpine tourism during the winter holidays resulted in a surge of COVID-19 cases in the Scandinavian countries, as many citizens brought back the virus after their return from their holidays in Italy and Austria [16]. Along the same line of reasoning, Gössling et al. have investigated the influence of tourists moving from China to other countries, while comparing the resulting COVID-19 spread with previous pandemics, also with a special focus on the impact it had on economies [17].

While we refrain from expressing specific positive/negative comments on these studies, we have to admit that the issue of how reliable and precise the predictions on the new infections as a function of tourist flows are remains crucial. Our general impression is that many of those studies reveal the weakness of drawing on mathematical models that are linear by nature, while the subtle implications of this phenomenon are probably due to non-linear dynamics, which has led to our effort to also experiment with a neural network, capable of capturing this kind of non-linearity.

We conclude this section by reporting on two other papers that are inspired by different visions of this problem. One of these two belongs to the group of simulation studies that are more interested in finding out how the virus spreads or decays, with the precise goal of guiding policy makers, yet without a meticulous attention to the precision with which they predict how many people are getting infected. To this group belongs the study conducted by D’Orazio et al., who investigated how certain COVID-19 containment measures could be more effective than others in limiting the spread of the virus in touristic cities, with the intent of going back to a *business as usual* regime, being pressured by economic reasons [18].

We cannot conclude without touching on the Susceptible-Infected-Removed (SIR) models and their derivatives, as those are the most popular modeling tools for epidemiology. These models are based on differential equations that describe the dynamics of the epidemic and need to calibrate their parameters on data in order to work correctly. Unfortunately, as Roda et al. have shown, there may not be enough data available, as the contagion unfolds, to calibrate them correctly and, more importantly, the more complex the model is (with an increasing number of parameters), the more this calibration becomes unmanageable [19]. It is therefore no coincidence that, while there have been numerous applications of this kind of models to the study of the spread of COVID-19 in general, there seems to be almost no work that integrates tourism and free movement of people using these models, as this would probably result in too much complexity to manage, as we were alluding to before.

However, an important consideration is due at this point: our work, like most of the other works we have cited on this specific subject, is based on tourist data from the past year because the current data is not yet available. As a consequence, all the predictions and considerations have been made starting from the assumption that the domestic tourist flow in 2020 can be considered comparable in number to that of the previous year, 2019. The same goes for the other factors that we considered, such as population density and healthcare expenditure, for example, which are, however, less likely to have drastically changed from 2019 to 2020.

The remainder of this paper is structured as follows. In the next section, we describe more precisely the data we used, as well as their sources and the methodologies we employed. Section 3 presents the results we obtained and discusses them. Lastly, Section 4 concludes the paper, presenting our final considerations.

## 2. Materials and Methods

In this Section, we will provide a description of the data on which our analysis is based, along with the methods used for its collection and the sources from which we collected it (Section 2.1). Then, we will present the methodologies we have chosen to conduct our various analyses (Section 2.2).

### 2.1. Data Collection

The first remark of interest for readers is that all the data we used is publicly available for consultation and can be downloaded from the websites of the Italian Institutions we mention below.

#### 2.1.1. COVID-19 Data

COVID-19 data is provided by the Italian Civil Protection Department (Dipartimento di Protezione Civile) that created a GitHub repository, which is updated daily [4]. This repository contains multiple counts, such as new infection cases, total cases, number of hospitalized cases, number of recovered patients, and so on. These can be aggregated on a national basis, per single region, or per single province.

**Figure 1:**
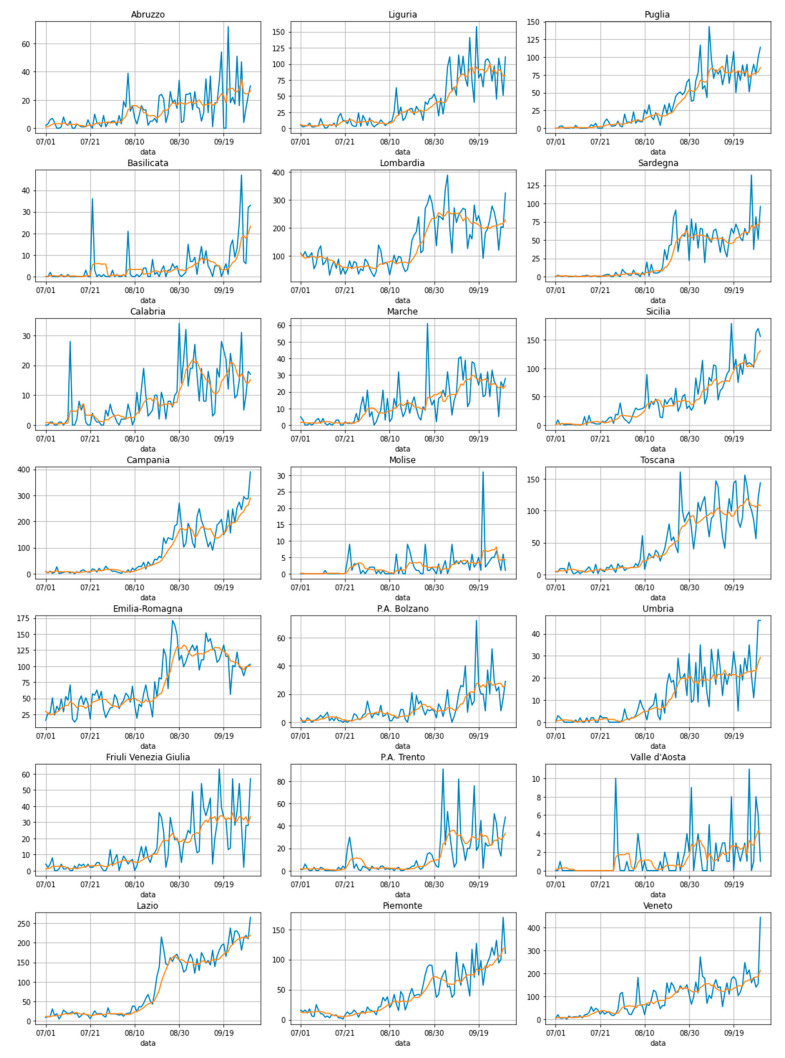
Number of new daily infection cases (in blue) and correspondent seven-day moving average (orange) for each of the 21 Italian regions, in the period between 1 July and 30 September. All data was collected from the dedicated GitHub repository created by the Italian government (https://github.com/pcm-dpc/COVID-19).

The dependent variable that has played the most important role in all of our mathematical elaborations is obviously the number of new daily infection cases, counted on a regional basis and spanning the period from 1 July to 30 September; a clear visualization of how this variable evolved with time is reported (in blue) in Figure 1.

To be noted is also the fact that, given the special nature of the process through which this data was collected, the number of daily infection cases can oscillate quite a lot, especially around weekends. For this reason, we decided to also take into consideration a seven-day moving average (it too is represented in the aforementioned Figure 1 in orange).

A final note concerns the exact number of Italian regions. There are 20, including the Trentino-Alto Adige; however, this region has recently been split into two different and autonomous provinces for administrative purposes (precisely: the Provincia Autonoma di Trento and the Provincia Autonoma di Bolzano). For this reason, the number of plots reported in Figure 1 are precisely 21. In other parts of this paper, instead, to make our counts easier, these two provinces have been reunited (summing up the numbers coming from both provinces); this was only for the scope of this paper does not change the general meaning of our study.

#### 2.1.2. Tourist Data

All the data concerning tourists was gathered by the Italian National Institute of Statistics (ISTAT), which delivers an annual survey on various statistics concerning tourism such as, for example, the number of incoming and outgoing tourists with respect to the various Italian tourist hospitality structures.

The important premise to be made is that, in the present study, we are going to use the tourist data for the year 2019 because the data for the current year are not yet available; this is under the assumption that the tourist flows in 2020 will be somewhat proportional to those of the previous year, albeit probably smaller in scale.

Another relevant issue, at this point, concerns the definition of what an incoming/outgoing tourist is, for each given region, and their respective roles in our study. Obvious is the fact that incoming tourists, for a given destination region, are those who travel to a region that is outside their permanent place of residence. Similarly, outgoing tourists, for a given region, are those who leave their place of residence in that given region to travel to a place that is not their permanent place of residence. For the purpose of our study, it is not enough to take into consideration only incoming tourists; the number of outgoing ones is also relevant as they could have left their region only temporarily to come back after a few days, bringing with them the infection caught elsewhere while travelling. All this highlights the fact that our more sophisticated models will take into account, for each region, both the incoming and the outgoing tourist flows.

**Figure 2:**
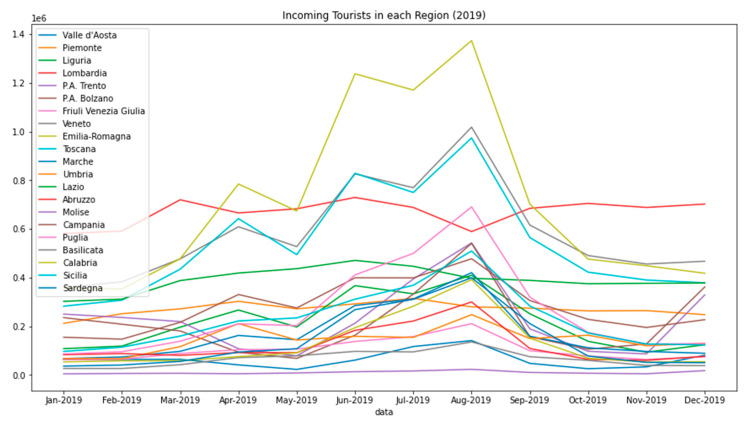
Incoming domestic tourists per each Italian region for each month of 2019. The *y* axis is arrivals per million. Typically, peaks of the curves are observed in August.

To give a visual impression of the volumes of moving tourists to the reader, Figure 2 reports this specific data: the incoming domestic tourist flows for each region in Italy over the entire year 2019, as made available in [10].

A first important fact to be noticed in Figure 2 is that all the peaks of the represented curves, counting the number of incoming domestic tourists for each Italian region, is situated in the month of August, this is a well expected result.

We encountered several difficulties in managing the available dataset of the tourist flows. For example, in the dataset we examined, there were two main indicators: *Arrivals* and *Presences*; the former is the number of individuals that arrive at each hospitality structure, the latter, instead, is the number of nights spent in that structure, both aggregated and on a regional basis. For the aim of this study, we decided to focus only on the so-called *Arrivals*.

Further, there would also be a further distinction between domestic visitors and visitors from abroad; again, we took the decision to count only the domestic flows, given the prevalence of this factor, owing to the various motivations we have already mentioned in Section 1 [6].

There was another final issue with this tourist data; while we can count on daily observations for the number of infected cases, all the tourist numbers, instead, are aggregated on a monthly basis. This poses a problem when one wants to investigate the relationship existing between the two different types of curves (i.e., new infections vs. tourist flows). To bypass this, there was no choice but to also aggregate, at the same scale, the number of new infected cases that, consequently, during the greater part of our investigations, were considered cumulatively, on a monthly basis.

Finally, putting all this together, the data model on which we have based most of our study can be summarized as described below.

We looked at the tourist data, per each region, as the total quantity of incoming/outgoing tourists of a one-month long sliding window that can shift one week at time, thus it can involve two different months at the same time. In that case, based on an assumption of a uniform distribution over time of the total number of incoming/outgoing tourists, we decided to utilize a linear combination of the quantity of the tourists from the two different months to yield the total number of tourists to be considered for that period.

A simple example will make it clearer. Consider a one-month long temporal window starting with the beginning of the second week of July and ending with the inclusion of the entire first week of August; this returns a total number of tourists to be taken into consideration, which is equal to the sum of 75% of the number of tourists for July plus 25% of the number of tourists for August.

**Figure 3:**
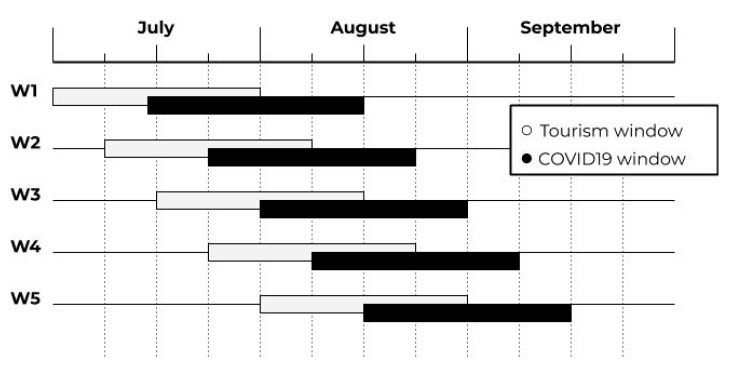
The tourism/COVID-19 dataset, structured based on sliding windows. White are the windows used for the tourist data, black are the windows used for the cumulative count of new infected cases. The black windows are shifted 14 days forward to account for the time required for the COVID-19 symptoms to manifest.

Figure 3 exemplifies this process, highlighting with the white windows, the count of the tourist flows. Obviously, this is not enough because, for each observation, the window with the number of tourists has to relate to the cumulative number of infected cases for that period, per each region.

This is taken into account in Figure 3, with the presence of the black windows that represent the cumulative number of new infections happening in that period. Those black windows can shift concurrently with the white ones, but with an initial offset of two weeks forward to take into consideration the fact that COVID-19 may manifest with two weeks of delay, after the initial day of the contagion.

In this way, we can shift our windows (white and black) one week at time over the entire subject period of our investigation, thus resulting in five different pairs of window (from W1 to W5 in Figure 3), with their relative data, per each region. If we count the 20 different Italian regions (considering Trentino-Alto Adige as a unique region), we yield a total of 100 pairs of windows along with the relative data to be used. The way we will use that data will be explained in the following Section 2.2.3 and Section 2.2.4.

#### 2.1.3. Other Relevant Data

Besides the tourist flows, in and out of each region, we decided to gather data relative to other additional variables that could play a role in this scenario. The rationale behind this decision was that, if we did not do this, we would be maintaining that only tourism is the factor that determines an increase in the number of new infections; this hypothesis is obviously quite far from reality. Thus, we also took the following data into account:

- The population density for each region (available for the year 2019);
- The annual expenditure on healthcare for each region (available for the year 2019);
- The percentage of population with more than 65 years of age (available for the year 2019);
- A categorical variable representing the geographical position of each region in Italy. ISTAT dictates that each region can belong to one of these following areas: North-West, North-East, Center, South, Islands.

As to the population density and to the percentage of citizens over 65 years old, the corresponding data was obtained from the ISTAT website [9]. The region classification, following the Nomenclature of Territorial Units for Statistics or NUTS (Nomenclature of Territorial Units for Statistics) convention, is reported in Table 1. Finally, the data on healthcare expenditures was obtained from the 2019 annual report, provided by the National Observatory on Health in the Italian Regions (Osservatorio Nazionale sulla Salute nelle Regioni Italiane) [20].

### 2.2. Methodologies

As anticipated in the Introduction, in our study, we first looked for evidence confirming that an increase of infected cases, in each Italian region, could be attributed to the domestic tourist flows of the summer of 2020. With this in mind, we analyzed the curves of the new infections to check if, during that summer, this phenomenon happened. To do this, we utilized the method described in Section 2.2.1.

Once this relationship (new infections/tourist flows) was confirmed, we tried to solve a consequent mathematical regression problem, which came as an extension of the aforementioned result: estimating, on a monthly basis, the total amount of new infections based on the number of inbound and outbound tourists, per region. The importance of this lies in the fact that finding a model that is able to solve this problem, with good accuracy, means acquiring the capability to predict in advance how the spread of COVID-19 progresses with tourists moving across the world. Section 2.2.2, Section 2.2.3, Section 2.2.4 are devoted to illustrating the three different mathematical models we have utilized to solve this problem.

#### 2.2.1. A Changepoint Detection Method

While one could look at all the graphs in Figure 1 and conclude that an uptick in the number of new daily cases is clearly visible around the end of August for most of the observed regions, our scientific duty was instead to validate this intuition and hopefully deliver more precise date (or point) as to when this change occurs.

To do that, various statistical methods could be applied to the time series of the graphs of Figure 1. The idea at the basis of all those methods is, generally, that of computing a kind of discrepancy measure between different parts of the observed time series. If the two parts do not present a relevant change (for example, in their mean or in their variance, or both) this discrepancy factor will be low, otherwise there will be a peak, and a change point will be precisely detected.

To find this change point in all the time series represented in all the 21 graphs of Figure 1, we used the change point selection method described in [21]. This is essentially a window-based method, whose algorithm works by calculating a discrepancy measure, d, based on the following formula:

(1) $$d\left(y_{\left\{u‥v\right\}},\;y_{\left\{v‥w\right\}}\right)=c\left(y_{\left\{u‥w\right\}}\right)-c\left(y_{\left\{u‥v\right\}}\right)-c\left(y_{\left\{v‥w\right\}}\right)$$

where *y*, in our case, is a cumulative count of infected cases and *u*, *v*, and *w* represent those days that delimit the boundaries of two temporal contiguous windows. More precisely, with *u* < *v* < *w*, we have two temporal windows; the first window spans from day *u* to day *v* and the second one from day *v* to day *w*. Obviously, these are two contiguous windows; hence, if we reunite them, we yield a unique temporal window, spanning the period from day *u* to day *w*. The cost function, *c*, utilizes the square of the L2 norm, and it is defined as follows:

(2) $$c\left(y_{I}\right)=\underset{t\in I}{\sum }\|y_{t}-\bar{y}{\|}_{2}^{2}$$

where I is the above mentioned temporal window and y, again, is the cumulative count of the infected cases, while $\bar{y}$ is the correspondent mean value computed over that considered window.

To individuate each change point for each of the Italian regions under observation, we looked for the maximum value taken by d in each region. All this clearly indicates the presence of a unique change point for each examined region in our study.

**Figure 4:**
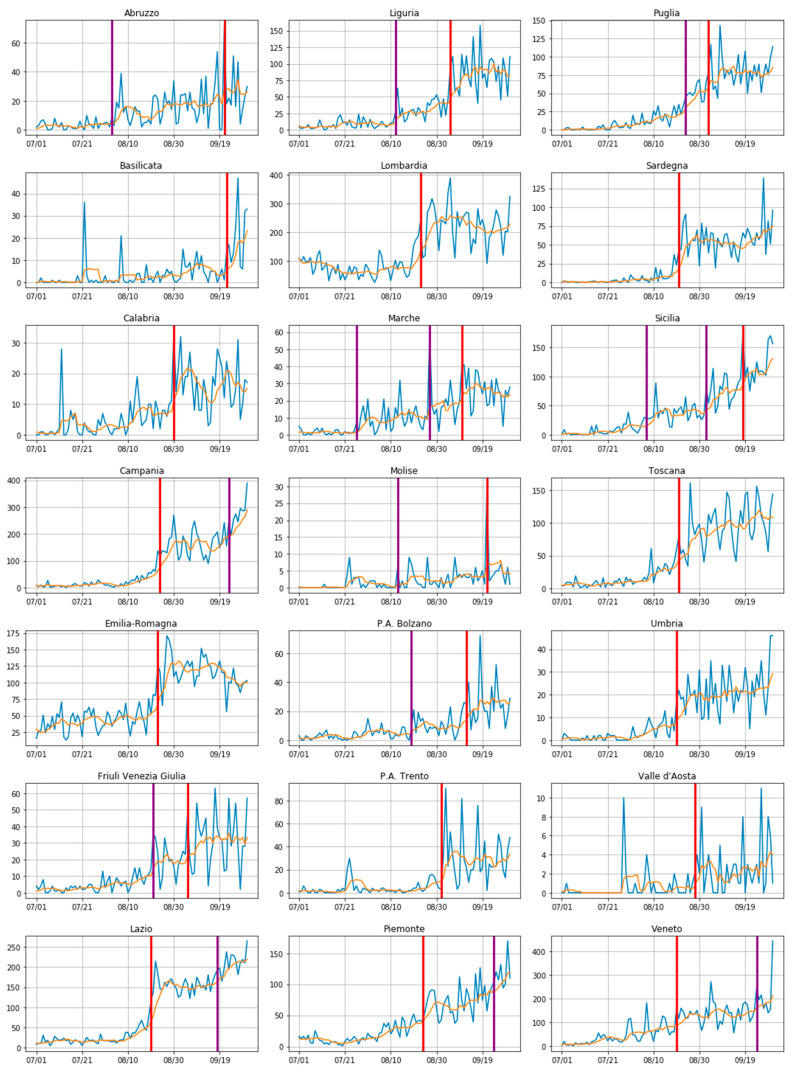
Change points (red lines) found by the change point detection method described in Section 2.2.1. In purple are all the change points detected with a variant of the method. The daily infections curve is depicted in blue, while the correspondent seven-day moving average is in orange. Period of observation: 1 July–30 September, for each of the 21 Italian regions.

The results are discussed, at length, in the Results section (where the change points are highlighted with red lines in all the plots of Figure 4). Here, is sufficient to say that all these points were clearly localized at the end of August (except for a couple of the smallest regions in our investigation).

Because the above window-sliding algorithm may represent a fast approximation of alternative optimal methods that could individuate multiple (or even no) change points, we have developed an additional experiment aimed at verify the validity of our method with a more complex approach based on the concept of threshold or penalty and using a penalty based Bayesian Information Criterion (BIC), as suggested in [21]. This additional algorithm returned more change points for each plot of Figure 4 (marked in purple), yet it has confirmed that the change points discovered with our basic window-sliding algorithm were still included in those sets, as were those with the maximum discrepancy value.

**Figure 5:**
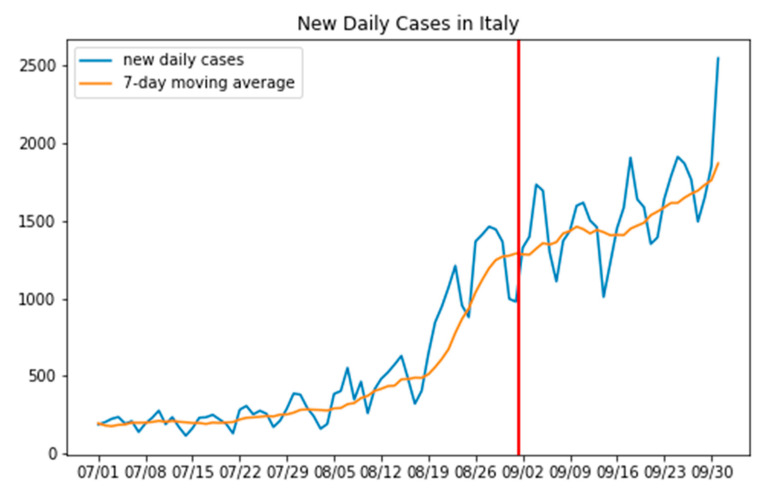
Number of new daily infection cases in Italy (blue), their 7-day moving average (orange), and the average national change point (red), falling on 1 September.

To give an impression of the general situation, the Results section also shows, in Figure 5, the mean value of when the change points occur if we consider the national case (red line in Figure 5). Nonetheless, it is to be noted here that this change point was simply computed as the average of all the change points that were found with the method discussed previously.

As seen in Figure 5, on a national basis, this average change point shifts just a little bit forward to 1 September. The following final consideration is necessary. It cannot be a coincidence that this date of 1 September happens around the two-week period taken by the virus to manifest itself. This date, thus, provides a first indicator of the fact that the spread of the virus may have been favored by the traditional peak in tourism, typically experienced in Italy around 15 August. Additionally, looking at the figure, we have some confirmation of the hypothesis of a relevant increase of infections occurring all over the country approximately 14 days after the occurrence, on 15 August, of the celebration of a special Italian holiday, called *Ferragosto*.

#### 2.2.2. A Simple Toy Model

To find a formula able to take into account the number of new infections that tourist flows can bring to each region, we began toying with a simple equation that leverages, per each considered region, the data relative to the distribution of incoming tourists from all of the other regions.

In essence, we started our activities with the simplistic idea that considers only that portion of possible infected tourists arriving at each destination region during August 2020, assuming that they end up infecting someone else, thus resulting in new infection cases.

The total number, *C*, of infected tourists arriving at each different destination region, i, is computed, first by multiplying the total number of tourists leaving a certain region, *j*, and arriving at that destination region, i, by the infection rate of the region, *j*, then by summing up all the contributions from all the regions by means of the following formula:

(3) $$C_{i}=\left(r+1\right)\cdot \overset{R}{\underset{j}{\sum }}T_{ij}\cdot I_{j},$$

where i and *j* are the two regions of interest (i is the destination), $T_{ij}$ is the number of tourists moving from the region, *j*, to the destination, i, and $I_{j}$ is the infection rate of region *j*. *I*, in turn, was estimated by calculating the ratio between the number of infected cases and the total population living in that region.

Finally, the multiplicative parameter, *r*, is a factor that should take into account how the contagion spreads as soon as the disease is diffused within each given destination region, thus resembling the so-called reproductive number of the SIR models [19]. In our case, considering the general Italian situation by the middle of August, we set *r* = 1. As to this particular choice for the value of *r*, we want to highlight the fact that it would not be possible to try and estimate it from the data, such as would be done in any normal statistical model, because there is actually no available public information concerning the number of infections, $C_{i}$.

We can anticipate that this methodology cannot, because of its nature, be too precise in its predictions, as there is no serious attempt to estimate any parameters from the data (we will see this fact clearly with the results in Section 3). Nonetheless, it has given us an initial, interesting perspective on the number of infections that could be ascribed to the presence of Italian tourists for each region. More important, here, is to note the fact that, to compute the aforementioned formula, we need to know how many tourists have arrived at a given region, i, while leaving a certain region, *j*.

Unfortunately, this kind of data, that is, the exact number of tourists that leave region *j* to come to region i, is not available from the data provided by ISTAT. Hence, just for this specific case, we resorted to a different source of information, which was a data repository from the Tuscany region where the pairwise cross-regional tourist movements are given for the year 2019 [22]. The technical problem here was that these flows are not given in terms of arrivals, but rather as the number of nights a given tourist from region *j* stays in the destination region, i. This problem was bypassed by assuming that, on average, all tourists stay overnight in a destination region for the same number of nights, away from the region they have left. This assumption has allowed us to obtain an estimate of the number of arrivals for each destination region that can be used in the above formula.

To conclude this section, we must also emphasize the fact that we have assumed an infection rate in the travelling population that is equal to that of the total population. This point could be questionable, as some classes of people could have traveled less or not at all. Nonetheless, the lack of more precise information on the demographics of the travelers made us decide not to add further hypothesis to our simple *toy model*.

#### 2.2.3. A Statistical Model with a Negative Binomial Regression

Let us now look at a more solid statistical model to solve the problem. We tailored a generalized linear model (GLM), assuming a negative binomial distribution for the number of new infections, whose parameters were obtained through maximum likelihood estimation. In this case, we judged the use of a negative binomial distribution as a desirable choice, owing to the necessity of counting data [15,23,24]. A possible alternative would be the employment of a Poisson distribution, which assumes equal values of mean and variance, making it unusable in the context of COVID-19 due to the well-recognized characteristics of over-dispersion manifested by the spread of this kind of disease, often referred to as a *super spreading* scheme [25,26]. In conclusion, the opportunity of using a negative binomial distribution is well recognized in the specialized epidemiological literature in the presence of viral spread phenomena, where the distribution of individual infectiousness is often highly skewed much like, for example, in the previous cases of SARS, MERS, and Ebola, in addition to COVID-19 itself. To simplify this discussion, the goal of this model is that of predicting, for each region, the cumulative number of new infections occurring during the period when one of the black windows, shown in Figure 3, is under consideration, displaying the result of the influence of the quantity of tourists visiting that region during the time of the white window that precedes the black window of interest. Further, given the characteristics of this model, other variables that could help to get more precise predictions were taken into account. The formula on which our negative binomial regression model is based is given below, following the formulation of [27]:

(4) $$\ln(E(Y_{iw}|T_{iw},D_{i},H_{i},O_{i},A_{i}))=\beta_{0}+\beta_{1}\ln\left(T_{iw}\right)+\beta_{0}\ln\left(D_{i}\right)+\beta_{3}\ln\left(H_{i}\right)+\beta_{4}O_{i}+\beta_{5}A_{i}.$$

where: *β*_0_, *β*_1_, *β*_2_, *β*_3_, *β*_4_, and *β*_5_ are the coefficients to be estimated, while:

- $Y_{iw}$ is the cumulative number of the new infection cases, occurring in a region, i during the time comprised within the (black) window, *w*;
- $T_{iw}$ is the sum of inbound and outbound tourists for a given region, i, during the aforementioned window, w;
- $D_{i}$ is the population density for each region, i, measured as the number of inhabitants per km^2^;
- $H_{i}$ is the healthcare expenditure for the region, i, expressed as a percentage of the region’s GDP;
- $O_{i}$ is the ratio between the total population and the population over the age of 65;
- $A_{i}$ is a variable that takes into account the area to which a given region belongs, as shown in Table 2;
- ln stands for the natural logarithm. We used logarithms at the right-hand side of the formula above, in accordance with the model proposed in [15], where this choice is mainly motivated by an increase in the performance prediction.

The results coming from the solution of this model will be presented in Section 3, where the predictions of the number of infected people will be delivered along with a comparison of those predictions to the number of the persons who actually got infected during that period in any given considered region.

Nonetheless, much of the interest in using this method relies upon its ability to properly estimate the involved coefficients, thus revealing how significant each of them is. To better discuss this issue, we present Table 2, which should be interpreted as follows.

Under *Coefficients*, the reader can find the names of the predictors whose coefficients need to be estimated. Under *Estimates* one can find the estimates of those coefficients, as per Equation (4). Along with those estimates comes an attempt to measure the errors made by the model when those estimates are computed. More precisely, the third column (*Std Error*) presents the standard deviations associated with those estimates, while the fourth column provides the so-called *z*-*value*, which is a statistic that returns a result different from the hypothesis, which is that the given coefficient is equal to zero. Put simply, when *z* is high (either positive or negative), there is a low probability that the coefficient under consideration is zero; that is, not relevant for the solution of the regression problem of interest. Finally, we come to the fifth column of Table 2, where the probability that the estimates set under the column *Coefficients* can exceed the modulus of the already explained *z*-*value* is estimated.

This last value, in particular, should be considered for an immediate analysis of the table, where the lower values under column 5 are associated with the most relevant predictors.

All this being said, what is quite interesting in this discussion is the careful observation of the estimates computed for the coefficients of the following predictors: tourism and density of population (*T* and *D*).

These two factors appear as important predictors with the capability to have an influence on the solution of the regression problem we are facing, as confirmed by their high significance, given that the estimates of their relative coefficients come with good precision, as an analysis of columns 3, 4, and especially 5, reveal.

It is also interesting to note how the Island and Southern regions seem to be linked to an increased number of infections (with respect to other Italian regions) because of the same statistical motivations we have explained before. Also of note, for similar reasons, is the predictor associated with the percentage of persons aged over 65 (*O*), while other predictors (e.g., healthcare expenditure, *H*) do not seem to play a relevant role for this model.

In summary, this model appears to be more influenced by how many infections are brought by tourists; the percentage of people with an age over 65 is of secondary importance, followed by how much money a given region spends on healthcare.

#### 2.2.4. A Cognitive Model

The final model with which we experimented was an Artificial Neural Network (ANN). In particular, we designed and implemented a feedforward neural network, sometimes referred to as a multilayer perceptron (MLP). The rationale behind this choice was to verify whether a cognitive model would be able to outperform the results returned by a classical statistical linear regression model (such as the GLM in Section 2.2.3) by picking up some non-linear relationships in the data that would not be evident to our intuition. This cognitive model was developed using the *Keras* library, part of the *Tensorflow* framework for deep learning [28].

We have had other previous experience with this kind of model, both to investigate how the COVID-19 spreads and we have also utilized it in other fields [29,30,31,32,33]. Here, the first fact to note is that we input to our ANN the same dataset, comprised of white and black windows (with relative numbers of infections and tourists), that was used with the GLM. Just a few minimal variations (not reported here, for the sake of conciseness) were needed to obtain a standardized numerical input that allowed our ANN to work properly.

With regards to the 100 pairs of windows discussed in Section 2.1.1, we used 80 of them as learning examples for our ANN, while 20 of them were utilized for the testing activity. In essence, we used all the white and black windows (and relative data), from W1 to W4 in Figure 3, for the training activity, while the white and black windows, denoted as W5, were used for the testing.

The ANN took the same exact nine predictors of the aforementioned GLM as input features. Specifically, our ANN was, thus, comprised of three layers, each with a number of neurons, as reported in Table 3, plus a ReLU activation function and a final single neuron that returns the result. The model was trained for 150 epochs with a batch size of 1, using the RMSProp optimizer. Each layer also features a L2 regularization [34].

As is evident from the description above, this ANN is neither excessively deep nor wide as, given the characteristics of the datasets with which we worked, an increased number of neurons and relative parameters would not be useful.

It is now important to count the number of parameters elaborated at each level of the ANN. This count is reported in the third column of Table 3. Simply put, at the first highest layer, the number of neurons, plus 1, has to be multiplied by the number of parameters initially available, which are the same 9 indicators that were used in the previous GLM of Section 2.2.3. This gives the number of parameters involved at that first level. With each lower level, instead, the number of values (plus 1) returned by the neurons of the highest layer has to be multiplied by the neurons comprised at that layer. Again, this gives the number of parameters managed by the ANN at that lower level.

Results coming from this cognitive model will be made available in the next Section; nonetheless, we can predict that the precision of the predictions we got increases with this cognitive model.

## 3. Results

We are now going to illustrate and discuss all the results we have obtained in our study, using the data and the models introduced in the previous sections.

### 3.1. Where the Infection Curve Starts to Change

Before moving to the performances of the three models, we intend to predict the number of new infected people as a function of the tourist flows, along with other relevant factors during the summer of 2020 in Italy; we would like to express a few thoughts on the results returned by the change point detection technique illustrated in Section 2.2.1. Here, those results are finally presented under the form of 21 different plots (Figure 4).

As anticipated, the most likely change point for the greater part of the infection curves of the Italian regions falls around the end of August (red lines in the figure), thus, the peak of the spread of COVID-19 in those regions is around the middle of that month (owing to the well-known 14-day lag). This period in time is exactly when most Italians usually go on holiday.

There are, however, a few exceptions, namely Abruzzo, Basilicata, Molise, and the autonomous province of Bolzano, whose change point is found to be closer to the middle of September. Nonetheless, it is important to note that those regions, being small in size, typically receive flows of tourists more gradually, never exceeding large volumes. Hence, the reason for that shift in time could lay there.

We would also like to point out that our detection method, because of its nature, has a harder time when called on to detect changes if they occur slowly and gradually, such as in the case of Sicily or Marche, for example. Further, as already anticipated, we have also run an additional experiment, using a change point detection method more complex than the one that returned the red lines highlighted in Figure 4. Again, this experiment has confirmed the validity of our initial approach, yet with more change points detected (purple lines in Figure 4). Of note is that none of those new (purple) change points exceeded, in terms of the value of the maximum discrepancy, *d*, the value of the red ones.

In conclusion, we can consider that our goal to obtain a data-driven description of how the infection curves evolved with the passage of time during the 2020 summer in Italy has been achieved, with the advantage that a significant change in those curves has been detected. The change is set typically around the end of August, as also the average national change point presented in Figure 5 confirms, thus corroborating the hypothesis that, in almost all the regions, the new infection cases started ramping up a couple of weeks after the tourism peaks (middle of August).

### 3.2. Predicting How Big This Change Is

We here come to the results we obtained from our three predictive models, namely: Simple Toy, Statistical with Binomial Regression, and Cognitive.

Table 4 illustrates all the results for each model. Some preliminary explanations regarding Table 4 are in order.

The first column of the table reports the number of real new COVID-19 infection cases, as recorded in the period between 15 August and 15 September. The second, third, and fourth columns in the Table report instead the number of infected people as predicted, respectively, by our three models: Simple Linear, Statistical with Binomial Regression, and Cognitive.

In Table 4, to emphasize which methodology was more able to return predictions closer to the real values, we have highlighted for each region (in red) the method that returns the *least inaccurate* prediction in terms of the smallest absolute error committed by the corresponding model (Simple Toy, Negative Binomial, and Cognitive) on that prediction.

Let us now start our discussion concerning the results returned by the Simple Linear model of Section 2.2.2.

It is important to remember here that the model only predicts new infections on the basis of the tourist flows, without any attention to other parameters. Hence, the absence of consideration for anything beyond those tourist flows makes it imprecise, yielding a general underestimation of the number of new infections (with the exception of Valle d’Aosta and Trentino Alto Adige). A further and final consideration on this simplistic model is that it underestimates the infections, especially in Lombardy, Lazio, Tuscany, and Campania. This may be due to the fact that we only considered domestic tourism, while those regions, especially their main cities (Milan, Rome, Florence, and Naples) are much visited by foreign tourists.

In all the other regions, this simplistic model sets the volume of the predicted infections, on average, as being half as much as the real number. In conclusion, it is worth stressing again how this toy model does not consider all those factors that could be relevant to a precise comprehension of this complex phenomenon.

Coming now to the negative binomial regression model of Section 2.2.3, we can see that the predicted infections are slightly more accurate, yielding nine different predictions (marked in red in Table 4) that are less inaccurate than those returned by the other models. This is especially true for smaller regions, where the use of other factors (besides tourism) that were taken into account by this model have likely brought a positive contribution in terms of the prediction capability.

Lastly comes the cognitive model (i.e., the ANN) of Section 2.2.4. This model has a predictive performance quite similar to those of the negative binomial regression model, with eleven different predictions (marked in red) that are less inaccurate than those returned by the other models.

**Figure 6:**
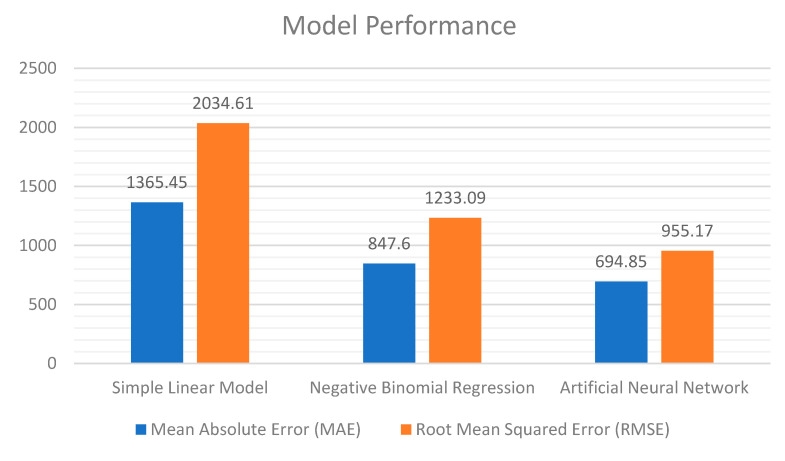
Model predictive performance: Mean Absolute Error (MAE) and Root Mean Squared Error (RMSE).

In addition, the cognitive model is the one that has made predictions, in general, with errors of a smaller magnitude compared to the others, probably because of the non-linear nature of its underlying neural network. This is confirmed by the histograms in Figure 6.

In Figure 6, we use the following metrics: The Mean Absolute Error (MAE) and the Root Mean Squared Error (RMSE). Simply put, the MAE represents the average error committed by the model in its predictions (regarding the difference between the real value and the prediction), as computed by the following formula:

(5) $$\mathrm{MAE}=\frac{1}{n}\;\overset{n}{\underset{i=1}{\sum }}\left|y_{i}-\hat{y_{i}}\right|,$$

where, in this case, n is the total number of predictions, $y_{i}$ is the $i^{th}$ real value, and $\hat{y_{i}}$ is the corresponding predicted value.

Instead, RMSE is the square root of the mean squared error, computed on the basis of the following formula, with the same meaning as above, for all the involved variables:

(6) $$\mathrm{RMSE}=\sqrt{\frac{1}{n}\;\overset{n}{\underset{i=1}{\sum }}{\left|y_{i}-\hat{y_{i}}\right|}^{2}.}$$

Put simply, Figure 6 highlights the fact that the cognitive model commits errors of a lesser relevance compared to the other models. For example, its predictions yielded an MAE of approx. 650 infections, while the model based on the negative binomial regression had an MAE of approx. 850 infections, thus confirming what we have already anticipated.

Nonetheless, it is clear that both models leave room for improvement. This is confirmed by a further measure, returned by the so-called Mean Average Percentage Error (MAPE) metric, which is obtained by putting the absolute error to the observed variable, in percentage terms, into a relation based on the following formula (where, again, the various variables have the same meaning as above):

(7) $$\mathrm{MAPE}=\frac{1}{n}\;\overset{n}{\underset{i=1}{\sum }}\left|\frac{y_{i}-\hat{y_{i}}}{y_{i}}\right|\times 100$$.

Based on this MAPE metric, we would see that our cognitive model returns a value of 36%, while the model based on negative binomial regression yields a value of 40%. Obviously, the smaller the percentage, the more accurate the model. Neither model has an MAPE value that can be considered satisfactory from this perspective. What is important, nonetheless, is the following consideration. If we think of the total number of infections that each region has suffered in the observed period; that is, those shown in the first column of Table 4, we have also to remember that not all those infections can be attributed to tourist flows as they represent the total number of new cases, which could have been caused by a variety of different factors. This shows quite well why the first simple linear model results were so inaccurate, as it was solely targeted at predicting the number of new infections resulting from tourism.

As for the other two models (Negative Binomial and Cognitive), while it is true that their learning activity relies on various other data beside tourism, for example, sanitary expenditure and age of the population, the fact cannot be hidden that many other factors, not included in the model, could exist that could have an influence on how the contagion spreads. Nonetheless, for many of them (think, for example, about the local reopening of public facilities or schools in September) the unavailability of reliable data makes it difficult for them to be considered, even now. Additionally, considering that many of these factors and their relative influence is unknown, we can conclude that the prediction inaccuracy of those two models is not enough to definitively disqualify them. Instead, they should be considered as a relevant starting point for the study of this phenomenon, if they could be further developed up to a point where they become able to capture all the various and complex spread patterns of this disease.

## 4. Discussion and Conclusions

In this paper, we set out to investigate the relationship between the COVID-19 spread and summer tourism across Italian regions. Our hypothesis was that the uptick in the number of new daily cases that every region, in one way or another, exhibited at the beginning of September 2020 was linked to the summer holydays that, in Italy, traditionally happen in August.

To cite the famous economist Tyler Cowen, as he told the Italian newspaper Il Foglio, “[…] *for many months you Italians did better than the dysfunctional Trump administration when it came to coronavirus. But now many European countries have lost that advantage, and this is because too many people wanted to travel and go on vacations in July and August*” [35]. After a peak of tourist movement around 15 August, the usual highpoint of the season, there would be an upshift of new infections about two weeks later.

This was the hypothesis and intuition we decided to test by using a statistical method able to detect a change in a time series. We went for a window-based approach that tested the discrepancy between different parts of the time series while looking for significant differences in the mean values of the new infections. The change points we found were, for the vast majority, located in the span of a few days around the end of August (and at the beginning of September), so much so that the mean change point for all regions falls on 1 September, thus providing a confirmation of our initial idea.

Nonetheless, as to this method, we want to highlight the fact that it only points to the maximum of this discrepancy. Working with a threshold or penalties would be an alternative solution, yet this would entail reasoning a value to assign to that threshold. In the end, for the sake of simplicity and interpretability, we opted for the present method. Nonetheless, we have also developed an additional experiment, based on a BIC scheme, that has confirmed our findings.

Before discussing the potential/limitations of the three subsequent models we proposed in this paper to better study the relationship between tourism and COVID-19, a few words on the dataset are in order.

The first thing to note is that COVID-19 count data can be very noisy (it exhibits a kind of weakly seasonality because of how the swabs are collected and registered); nonetheless, our decision to use a moving average has helped to regulate things, as shown in Figure 4. Furthermore, our choice to use a window-based method to analyze the evolution of the daily infection cases further allowed us not to suffer from the influence of this kind of problem.

The tourist data, however, is the thing that has to be considered as having the most limitations. The most obvious of these is the fact that we have worked with data from 2019, assuming that 2020 tourist flows (currently unavailable) are somewhat proportional. It should be clear that we cannot validate this supposition until this information is made available for the current year, 2020. Second, while the number of tourists that registered at holiday accommodations is a good proxy for cross-regional flows, it also is very limited if we try to take it as a precise description of how people have moved.

We now come to our three models.

As to the first one, which was based on a simple linear model, we must admit that it was a first and simplistic experiment, carried out with the aim of getting a very preliminary and general impression of what could be extrapolated from the data regarding the distribution of incoming tourists for each region. The idea was that combining the infection rate of every region with the number of tourists that exited that area would have given us a rough measure of the infection brought by tourism to each destination region. The limitation of this approach is clearly its lack of precision, as the only factor it considers is the number of incoming tourists; nonetheless, this simplicity also brings with it an interesting consideration: if we just look at the imported infection cases, the message is that, in many cases, up to the 50% of the infections occurring in August could have been avoided, or at least contained, if only the guard was not let down for the summer holidays.

The second approach, instead, had a two-fold goal: that of creating a model that one could manage using regression analysis and that made available reasonable predictions on the number of new infections. To achieve this, we had to integrate in our regression other relevant factors besides tourism because, while tourist flows have an influence on the total number of new infection cases, they cannot be considered the only reason why COVID-19 spreads. The main result, here, has been that using aggregated data on tourist flows along with other predictors such as population density and healthcare expenditures (as suggested by other works in the field) we managed to create a model that was able to make acceptable predictions. More importantly, our regression analysis confirmed that tourism was indeed a relevant predictor for the number of new infections, especially for the insular and southern regions in Italy, which are, typically, the most appealing regions for summer vacations. To be cited as a technical limitation of this model is the fact that, given the current state of the available tourist data flow, we could not increase the number of additional predictors and integrate them into the model as this would yield an number of parameters greater than the number of observations in the tourist dataset.

We would like to devote the last comments to the third model. Our intention was to experiment with a more modern and sophisticated cognitive method, an artificial neural network, with the hope that this would perform better in the prediction task. The main strength of these cognitive models is their capability to capture the complex non-linear relationships of the underlying data, even though this is counterbalanced by their weakness of needing a considerable amount of data to do so. Unfortunately, while some data, such a sanitary information, is quite abundant, the same cannot be said for tourism and similar contexts [36,37].

Nonetheless, our results have confirmed the expectation that a cognitive model, such as the one we employed, has the potential to make more precise predictions than a linear regression model, yet only to a limited extent in our specific case due to the limitations imposed by the available data.

To conclude, we have shown how tourism has had an impact on the spread of COVID-19 in the Italian summer of 2020. This result also confirms a trend that has already been observed in studies conducted to analyze other countries.

Finally, we believe that the two main takeaways are that policy makers should be wary of the dangers connected to unrestricted tourist mobility and gatherings during the pandemic, because lowering the guard has led to the current rise in cases, and that scientific efforts in tracking the contagion spread should consider tourist mobility as an important factor in predicting the risk of future outbreaks as long as tourist travel is allowed.

## Figures and Tables

**Table 1:** Italian regions distributed over the five areas.

Classification	Regions
North-West	Valle d’Aosta, Piedmont, Lombardy, Liguria
North-East	Veneto, Emilia-Romagna, Friuli-Venezia Giulia, Trentino Alto-Adige
Center	Tuscany, Lazio, Umbria, Marche
South	Campania, Abruzzo, Molise, Puglia, Basilicata, Calabria
Islands	Sardinia, Sicily

**Table 2:** Coefficient Estimates for our generalized linear model (GLM). They show how tourism (T) and density (D) are highly significant, as well as the geographical indication (A) for Island and Southern regions (albeit less). The percentage of elderly (O) is also included.

Coefficients	Estimate	Std. Error	*z*-*Value*	Pr(>|z|)
(Intercept)	−9.93064	2.10982	−4.707	2.52 × 10^−6^
log(T)	0.85395	0.12426	6.872	6.31 × 10^−12^
log(D)	0.82921	0.14986	5.533	3.14 × 10^−8^
log(H)	−1.19588	0.59292	−2.017	0.04370
O	11.12581	3.68649	3.018	0.00254
(A) Islands	1.33754	0.35277	3.792	0.00015
(A) North-Est	0.06887	0.17690	0.389	0.69705
(A) North-West	−0.31216	0.19199	−1.626	0.10397
(A) South	0.65365	0.27646	2.364	0.01806

**Table 3:** Structure of the Artificial Neural Network (ANN). Total number of parameters is 377, as the result of the sum of 160 + 136 + 72 + 9.

Layer (Type)	Neurons	# of Parameters
layer_1 (ReLU)	16	160 (16 × 10)
layer_2 (ReLU)	8	136 (17 × 8)
layer_3 (ReLU)	8	72 (9 × 8)
output	1	9 (9 × 1)

**Table 4:** Cumulative number of new infection cases as predicted by different models. Period: 15 August–15 September. Values in red correspond to the model with the smallest absolute error on that prediction, with respect to the other models.

Region	Real Cases	Simple Toy Model	Negative Binomial	Cognitive
Abruzzo	480	270	463	293
Basilicata	135	101	91	117
Calabria	383	275	298	270
Campania	3959	391	1466	2007
Emilia-Romagna	3325	1430	2133	2484
Friuli Venezia Giulia	677	195	362	348
Lazio	4303	334	1619	2168
Liguria	1504	405	907	889
Lombardia	6239	571	3349	4329
Marche	542	379	513	470
Molise	84	18	37	101
Piemonte	1817	229	781	983
Puglia	1689	560	922	857
Sardegna	1452	395	491	610
Sicilia	1625	372	1254	1034
Toscana	2412	907	1314	1557
Trentino-Alto Adige	869	1105	472	778
Umbria	546	212	223	199
Valle d’Aosta	46	138	40	67
Veneto	3852	999	2252	2557
